# Highly Thermally Resistant Bisamide Gelators as Pharmaceutical Crystallization Media

**DOI:** 10.3390/gels9010026

**Published:** 2022-12-29

**Authors:** Iván Torres-Moya, Abelardo Sánchez, Basanta Saikia, Dmitry S. Yufit, Pilar Prieto, José Ramón Carrillo, Jonathan W. Steed

**Affiliations:** 1 Department of Organic Chemistry, Faculty of Chemistry, University of Murcia, 30100 Murcia, Spain; 2 Department of Organic Chemistry, Faculty of Chemical Sciences and Technologies, University of Castilla La Mancha-IRICA, 13071 Ciudad Real, Spain; 3 Department of Chemistry, Durham University, South Road, Durham DH1 3LE, UK; 4 Department of Chemical Sciences, Tezpur University, Napaam 784028, India

**Keywords:** bisamide, hydrogen bonding, organogels, drug crystallization, flufenamic acid

## Abstract

Three simple bisamide derivatives (**G1**, **G2** and **G3**) with different structural modifications were synthesized with easy synthetic procedures in order to test their gel behaviour. The outcomes showed that hydrogen bonding was essential in gel formation; for this reason, only **G1** provided satisfactory gels. The presence of methoxy groups in **G2** and the alkyl chains in **G3** hindered the hydrogen bonding between N-H and C=O that occurred **G1**. In addition, **G1** provided thermally and mechanical stable gels, as confirmed with T_sol_ and rheology experiments. The gels of **G1** were also responsive under pH stimuli and were employed as a vehicle for drug crystallization, causing a change in polymorphism in the presence of flufenamic acid and therefore providing the most thermodynamically stable form III compared with metastable form IV obtained from solution crystallization.

## 1. Introduction

The booming interest in supramolecular gels in the last decade contrasts with their initially slow development following their original discovery in the 1930s [1]. In particular, low-molecular-weight gelators (LMWGs) have aroused great interest and comprise organic or coordination compounds with a molecular weight lower than 2000 Da; LMWGs are capable of gelling organic solvents or water. The interest in LMWGs arises from their versatility, good solubility upon heating, and ability to gel at low gelator concentrations (often less than 1% by mass), as well as the gels’ thermally reversible sol-to-gel phase transition. Gels obtained from LMWGs are usually prepared by cooling a heated solution of LMWGs in the solvent to be gelled, leading to a supersaturated solution. This supersaturation induces a rapid assembly of the gelator molecules into elongated fibres of typically 5–100 nm in diameter; these fibres subsequently aggregate into a 3D entangled network, thereby turning the liquid into a supramolecular gel [2]. Hence, supramolecular gels consist of an entangled fibrous network of gelator molecules held together by specific intermolecular interactions between gelators. Despite the fact that there are many different LMWGs with very different structures, it is still a challenge to predict the molecular structure of a possible gelator and the solvents in which it might form a gel. In general, the self-assembly of gelators into fibrous networks is driven by non-covalent interactions, such as van der Waals interactions, **π**−**π** interactions, dipolar interactions, hydrogen bonding, or coulombic interactions. Furthermore, solvophobic effects also play an important role because they contribute to the gelating ability by reducing the overall solubility of a gelator in a specific solvent [3,4,5].

To be able to design new gelators or even to predict gelation behaviour from molecular structure requires basic insight into the physicochemical basis for their gelation behaviour [6]. The problem is that these systems and the structures of the gels themselves have a large structural diversity. Many different techniques have been applied to establish the intramolecular interactions involved in self-assembly leading to gelation in order to elucidate the structures and morphologies of gels and fibres and to determine their thermotropic and viscoelastic properties [7].

**Scheme 1:**
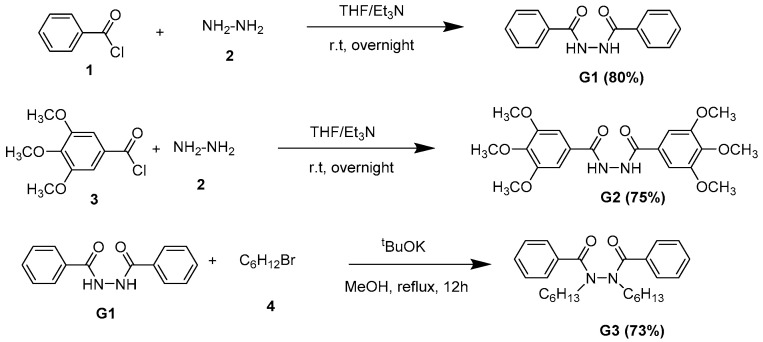
Synthesis of the possible **G1**–**3** gelators.

One emerging application of low-molecular-weight organogels in the last decade is as media for the crystallization of pharmaceuticals. The control of the solid form of a drug and its properties, particularly bioavailability, is crucial in the pharmaceutical industry [8,9,10]. The crystallization of the active ingredient is of vital importance because the polymorphic form, the crystal size, or the crystal morphology can influence its solubility, compressibility, melting point, bulk density, and dissolution rate [11,12]. In this sense, gels can be interesting media for polymorph discovery [13,14,15] and may offer interesting benefits such as the control of a specific polymorphic form, thus offering a potential alternative nucleation pathway compared with conventional solution crystallization. As a result, there is growing interest in organic compounds that have the ability to form gels and that can interact with a target pharmaceutical compound in such a way as to influence its crystallization outcome. However, the preparation of a gelator with appropriate functionalities can involve a complicated multi-step synthetic procedure. In this work, we describe the preparation of three simple bisamide-derived LMWGs (G1–3) (Scheme 1) and their application in pharmaceutical crystallization. It is anticipated that the presence of amide or methoxy groups may facilitate the formation of gels through hydrogen bonding [16]. Likewise, the presence of the alkyl chains in G3 confers flexibility and hydrophobicity to the molecule and facilitates the gelation process [17].

## 2. Results and Discussion

### 2.1. Synthetic Procedure

Compound **G1**, which was previously described by Shanmuga et al. for different purposes [18], was synthesized for the purposes of this work, and **G2** were prepared from acid chloride and anhydrous hydrazine [19]. Compound **G3** was prepared from **G1** through an alkylation reaction employing KOH and hexyl bromide (Scheme 1) [20]. All compounds provided satisfactory characterization data.

### 2.2. Gelation Tests

Gelation tests for compounds **G1**–**3** were performed with thirty different solvents and at different concentrations (2%, 1% and 0.5% wt.). Solvents were chosen to span the polarity spectrum from water to hexane and included aliphatic media; electron-rich and electron-poor aromatic solvents; and protic, dipolar aprotic, and halogenated systems. The gelators were dissolved in 0.5 mL of the chosen solvent followed by sonication for 30–60 s until complete dissolution and controlled heating at a temperature slightly below the boiling point of the solvent for a minute. Then, the vials were left undisturbed at room temperature and were tested for gelation at various time intervals: 30 min, 4 h, 24 h, 48 h and 72 h. Simple tube inversion was used to detect gel formation. The results are summarized in Supporting Information in Tables S1–S9.

**Figure 1:**
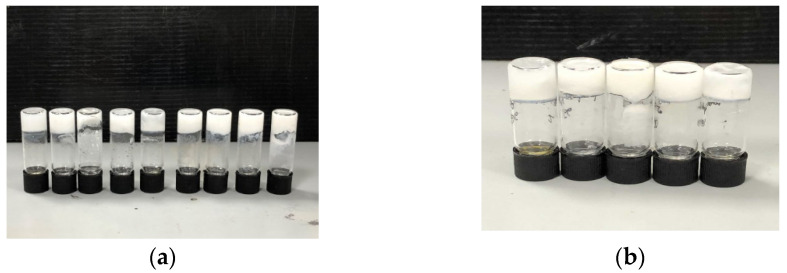
(**a**) Gels of **G1** at 2% wt. in (from left to right) dichloromethane, ethanol, water, 1-pentanol, 1-propanol, 2-butanol, acetone, acetonitrile and ethyl acetate. (**b**) Gels of **G1** at 1% wt. in (from left to right) dichloromethane, 1-pentanol, ethyl acetate, water and 1-propanol.

The results of the gelation tests showed that compound **G1** provided a range of gels, while **G2** and **G3** did not gel in any solvent. Gelator **G1** formed gels in nine different solvents (dichloromethane, ethanol, 1-pentanol, 1-propanol, 2-butanol, acetone, acetonitrile, water and ethyl acetate) at a concentration of 2% (Figure 1a) and in five solvents (dichloromethane, 1-pentanol, 1-propanol, water and ethyl acetate) at 1% wt. (Figure 1b). It remains very challenging to produce and rationalize gelation behaviour in particular solvents, and the solvents that formed gels here were those in which the compound had intermediate solubility that allowed for fibre formation when hot but resulted in insolubility when cold. This compound proved to be highly effective in the gelation processes, especially in polar solvents such as water, probably due to the presence of the bisamide group that enabled the formation of hydrogen bonding. In contrast, the presence of the aliphatic chains in **G3** avoided the formation of hydrogen bonds. **G2** showed a curious behaviour, since it did not form any gels despite having amide and methoxyl groups in its structure that could facilitate the formation of hydrogen bonds.

**Figure 2:**
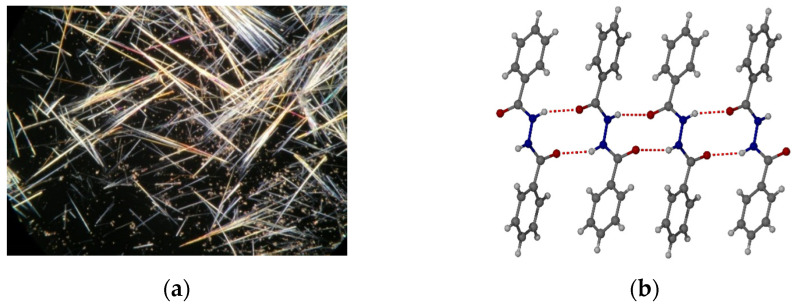
(**a**) Crystals of **G1** from ethylene glycol. (**b**) Crystal packing in **G1** showing hydrogen-bonded interactions between N-H and C=O groups (N···O 2.92 Å) [18].

**Figure 3:**
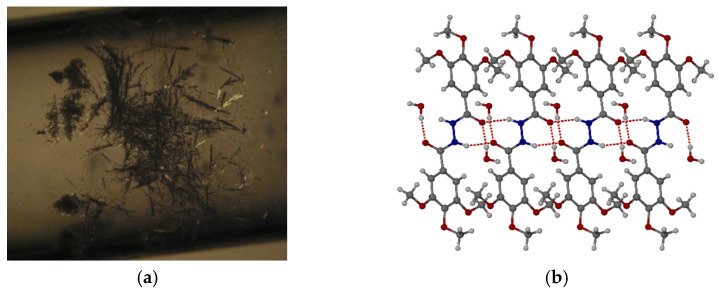
(**a**) Crystals of **G2** from ethanol. (**b**) Crystal packing in the dihydrate of **G2** crystallized from ethanol (N···O 2.9905(18), O_water_···O_water_ 2.7672(13); O_water_···O=C 2.7830(17) Å).

In order to probe the aggregation of the candidate gelators, the structures of **G1** and **G2** were determined with single-crystal X-ray diffraction. The crystal structure of **G1** was previously described by Shanmuga et al. [18]. Unfortunately, no suitable single-crystals of **G3** were obtained. In **G1**, the main interactions in the solid state structure were found to be hydrogen bonds between the N-H and C=O groups (Figure 2). Compound **G2** crystallized as a dihydrate from ethanol. The steric bulk of the trimethoxyaryl groups limited the ability of compound **G2** to form a hydrogen-bonded chain between the N-H and C=O groups, and the NH···O interactions were longer than in **G1** at 2.99 Å (Figure 3). The water of hydration formed an infinite chain linking one amide chain to another. The longer and hence weaker hydrogen-bonded chain interactions and the need to include water in the structure of **G2** may offer an explanation for its lack of gelation behaviour.

Critical gelation concentration (CGC) is an essential parameter for determining the capacity of a gelator to form a gel [21]. It can be defined as the minimum concentration of a gelator needed to form a consistent gel. These values are provided in Table 1.

The CGC for **G1** ranged between 0.6 in the cases of dichloromethane and ethyl acetate and 1.5 in the cases of ethanol and acetonitrile. The CGCs were found to be relatively low for very polar solvents such as ethyl acetate and dichloromethane or more hydrophobic alcohols such as 1-pentanol and 1-propanol.

The gel-to-sol phase transition temperature (T_sol_) was also determined for the gels of **G1** by employing the dropping ball method [22,23] and the results were recorded in Table 2. In this method, a small ball is deposited in the middle of a gel surface. Then, the temperature is slowly increased until the gel transforms into a sol and the ball touches the ground of the vial. The temperature at this point is T_sol_. The ball must be inert with respect to the gelator and not too heavy or too light to avoid the ball sinking into the gel or floating on the solution surface. Some gels of **G1** were found to be very stable, for example, in 1-pentanol, 2-butanol, and (especially) water or ethyl acetate, with values above the solvent boiling point in some cases. The strong hydrogen bonds might explain the thermal strength of these gels and the high values of T_sol_. Reductions in the concentration led to decreases in T_sol_ as expected, but in some solvents such as dichloromethane and 1-propanol, the stability was practically the same regardless of the concentration.

**Figure 4:**
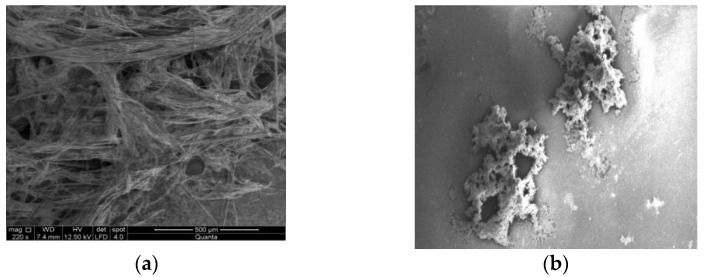
(**a**) SEM images for **G1** xerogels dried from dichloromethane. (**b**) SEM images for compound **G3**.

The morphology of the supramolecular aggregates in the form of dried xerogels were studied with SEM [24,25]. As usual, gels from **G1** showed an interconnected fibrous system [26] (Figure 4a). On the other hand, an amorphous morphology was observed for **G3**, which was consistent with the lack of gelation of this compound (Figure 4b).

To study the resistance under mechanical stimuli of the gels, we performed rheology experiments of gels in different solvents in which gels were formed at the same concentration (2% wt.) and compared the stability of the different gels for the same solvent at different concentrations. Frequency sweep experiments were performed and showed that in all the gels, the elastic modulus, G′, was at least an order of magnitude greater than the viscous modulus, G″. The experiments also showed that G′ was practically unchanged regardless of frequency, thus confirming the gel behaviour observed in the previously described inversion tube tests. The parameter that was used for the comparison was the yield stress (σ), which can be defined as the point which gels break down under mechanical stimuli and begin to shear. As a general rule, the stability of gels is higher with high values of yield stress [27].

**Figure 5:**
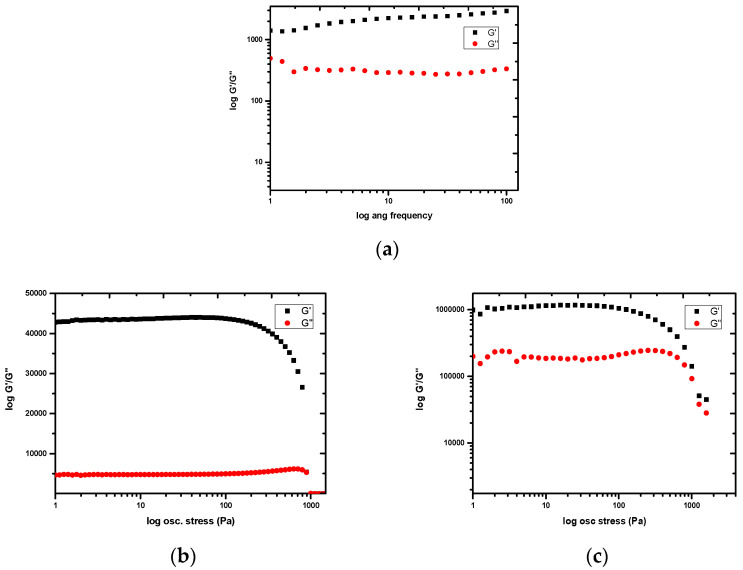
(**a**) Frequency sweep experiment at a concentration of 2% in acetonitrile. Stress sweep experiments at a concentration of 2% wt. for (**b**) **G1** in 1-pentanol (σ = 1000 Pa) and (**c**) **G1** in ethanol (σ = 1995 Pa).

The gels of **G1** proved to be highly robust, with σ values of more than 1000 Pa in 1-pentanol and ethanol, as shown in Figure 5. The low σ values in the case of water are remarkable, and the mechanical stability of the hydrogel was relatively low. The results of the remaining stress sweep experiments are described in the SI (Figures S1–S7).

In addition, gels in the same solvent (1-pentanol) were compared at different concentrations (2% vs. 1% wt.) (Figure 5b vs. Figure S8 in the SI). In the same way as the T_sol_ experiments, the mechanical stability of the gels (confirmed by the decreasing σ) decreased with the concentration because the number of interconnections between the fibres decreased due to the lower fibre density causing the weakening of the gel.

### 2.3. Stimuli Response

**Figure 6:**
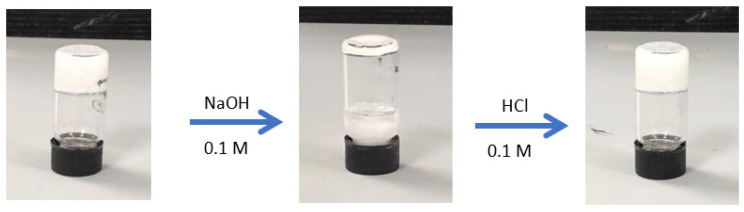
pH response of **G1**.

Gel responsiveness under different stimuli is of interest in the context of smart materials [28,29]. The importance of hydrogen-bonding interactions in the organogels of **G1** led us to study the influence of pH on its gelation process. **G1** gels in ethanol at a concentration of 2% wt. were treated with a solution of NaOH 0.1 M (pH = 13) to induce the transformation of the gel into a sol (Figure 6). The results of this treatment indicated the breaking of the hydrogen bond network due to the deprotonation of the bisamide group or hydrogen bond competition by the excess hydroxide ions. This hypothesis was formulated after the treatment of this mixture with a solution of HCl 0.1M (pH = 1). In this case, the gel recovered its initial structure (Figure 6), again indicating re-protonation and confirming the great importance of hydrogen bonds in the formation of **G1** gels.

### 2.4. Gel Phase Crystallization of Pharmaceutical Drugs

**Figure 7:**
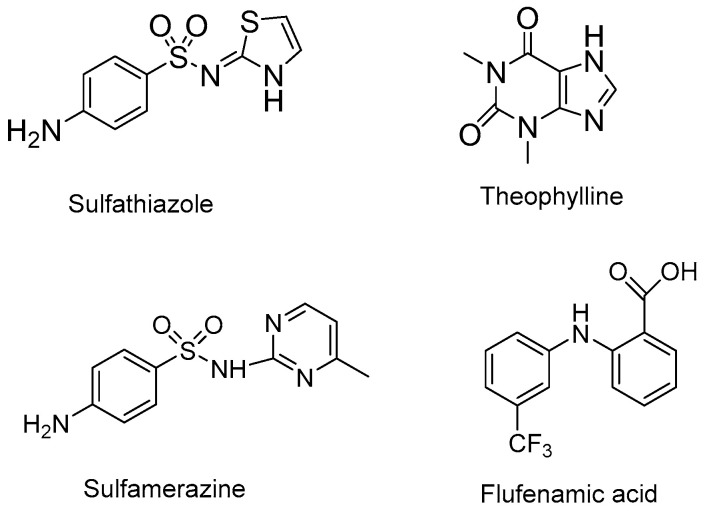
Chemical structures of the APIs chosen for crystallization in gels of **G1**.

Gels with efficient thermal and mechanical resistance can be applied in different applications as drug delivery or drug crystallization systems. Hydrogels are suitable for drug delivery due to their potential biocompatibility caused by the presence of water in their structures. Despite the fact that **G1** can form hydrogels, its low mechanical resistance confirmed by rheological studies indicates that this gel is not suitable as a drug delivery vehicle. However, gels of **G1** are potentially suitable for the crystallization of active pharmaceutical ingredients (API) [8,9,10]. Small-molecule organogels reduce convection (thus allowing for diffusional growth) and have the possibility of functional-group-specific interactions with the gel surface, potentially allowing the gel to act as a heteronucleation surface. Based on the structure of **G1,** we screened the crystallization of potentially complementary active pharmaceutical ingredients (APIs) in gels. We chose APIs with efficient NH-type hydrogen bonding functional groups, known polymorphism, and aromatic groups to potentially π-stack with the gel surface, namely, theophylline, sulfamerazine, sulfathiazole, and flufenamic acid (Figure 7).

**Figure 8:**
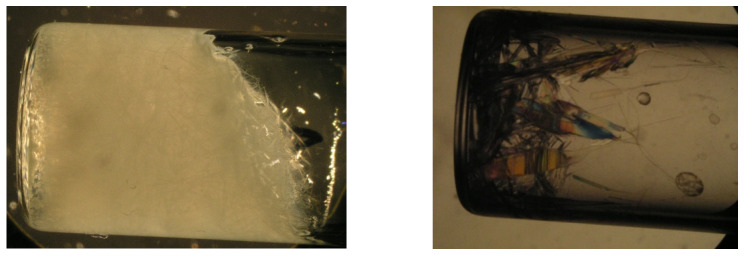
Crystals of flufenamic acid grown in dichloromethane gel of **G1** (**left**) and with solution evaporation (**right**).

A wide range of different crystallization tests were carried out to optimize the crystallization conditions of the APIs. The best gelling conditions were found when using a 10 mg/mL drug concentration at a 1% **G1** concentration. In a vial, a mixture of **G1** and the different APIs in a specific solvent was heated, sonicated, and kept undisturbed at room temperature for up to several weeks to allow crystallization to complete. In many cases, crystallization was not observed. The tests were repeated in the same conditions five times. The vials were visually checked for the presence of solid materials, and the resulting crystals were analysed with X-ray powder and single-crystal X-ray crystal diffraction. The **G1** gelator showed the ability to act as a successful host for the crystallization of all APIs; however, the same polymorphic form was observed in both a control solution and the gel phase crystallization for sulfathiazole, theophylline, and sulfamerazine. The outcomes showed the existence of kinetic form II in the case of theophylline in dichloromethane [30,31], the *Pna*2_1_ polymorph in the case of sulfamerazine [32], and polymorph II in the case of sulfathiazole [33,34]. On the other hand, in the gel crystallization of flufenamic acid, a change in the polymorphism from form IV in solution to form III was observed in the presence of the **G1** gelator [35]. The polymorphism was confirmed with the unit cell determination of the crystals (data in the SI). This change in the polymorphism was also accompanied by a change in the morphology of the crystals from block crystals in solution evaporation to thick needles crystals in gel crystallization (Figure 8) These results suggested that the presence of **G1** inhibited the crystallization of form IV and favoured the nucleation of form III of flufenamic acid, perhaps as a result of the common acid–amide heterosynthon [36]. Form III is the most thermodynamically stable form at room temperature, and it is created with stirred cooling of a hot toluene solution [37]. Its observation under conditions that favour a metastable polymorph suggests that the studied gels may be useful in sidestepping the formation of metastable forms to target the pharmaceutically desirable thermodynamic phase.

## 3. Conclusions

Three readily synthesized bisamide derivatives were tested as possible gelators. Compound **G1** proved to be an effective gelator with a wide range of gels in different solvents at different concentrations with response under pH stimuli. Some gels of **G1** exhibited high thermal and mechanical resistance. In addition, gels of **G1** were employed in the crystallization of theophylline, sulfamerazine, sulfathiazole, and flufenamic acid. In this last case, the presence of the gel induced a change in the polymorphism to the most thermodynamically stable form, form III, under ambient conditions compared with a control solution under the same conditions. These results suggest the potential of gels to ‘sidestep’ metastable forms and allow for the direct crystallization of the pharmaceutically desirable thermodynamic form.

## 4. Materials and Methods

All reagents were used without any previous purification. Reactions with materials sensitive to air were performed under an inert atmosphere of argon. Flash chromatography was performed with silica gel (Merck, Kieselgel 60, 230–240 mesh or Scharlau 60, 230–240 mesh). Analytical thin-layer chromatography (TLC) was carried out with the use of aluminium-coated Merck Kieselgel 60 F254 plates. NMR spectra were recorded with a Varian Unity 500 (^1^H: 500 MHz; ^13^C: 125 MHz) spectrometer at 298 K while employing deuterated solvents as internal references against the residual protic solvent signal. Chemical shifts (δ) are described in ppm. Multiplicities are denoted in the following way: s = singlet; d = doublet; t = triplet; m = multiplet; br = broad.

Melting points were determined with a Buchi Melting-point 565 instrument.

SEM images were obtained with a JEOL JSM 6335F microscope working at 10 kV.

In the drop-milling test used to calculate T_sols_, a small metal ball with a diameter of 1 cm was used.

Rheological measurements were carried out with advanced AR 2000 rheometer from TA Instruments that was equipped with a cooling system (Julabo C). A 20 mm plain plate geometry (stainless steel) was also employed. Firstly, strain sweep measurements were carried out in order to estimate the strain in percent at which reasonable torque values were given (about 10 times of the transducer resolution limit). Then, frequency sweep measurements and time sweep measurements from 0.1 to 4000 Pa were carried out.

Gel phase crystallizations were carried out with the addition of 10 mg of the drug in a vial containing 1% wt. of the **G1** gelator in a particular solvent. The vials were warmed and then sonicated to achieve a gel of the API solution. Crystals started appearing after a few days, as observed with microscopy. Crystals were characterized with the determination of the unit cell parameter.

The single-crystal X-ray data for **G2** dihydrate were collected at a temperature of 120.0(2)K using MoKα radiation (λ = 0.71073 Å) with a Bruker D8Venture (Photon100 CMOS detector, IμS-microsource, focusing mirrors) 3-circle diffractometer equipped with a Cryostream (Oxford Cryosystems) open-flow nitrogen cryostat. The structure was solved with the direct method and refined with full-matrix least squares on F^2^ for all data using Olex2 [38] and SHELXTL [39] software. All non-hydrogen atoms were refined in anisotropic approximation, and hydrogen atoms were found with difference Fourier map and isotropically refined. Crystal data and the parameters of refinement are summarized in the Supporting Information in Tables S10–S14. Crystallographic data for the structure were deposited in the Cambridge Crystallographic Data Centre as supplementary publication CCDC-2216521.

### Synthetic Procedures for the Synthesis of ***G1***–***3***

N’-benzoylbenzohydrazide (**G1**): In a two-necked flask, THF (20 mL), hydrazine (1.14 g, 35.71 mmol) and Et_3_N (20 mL) were added at 0 °C and under an inert atmosphere. Then, acid chloride (10.0 g, 71.42 mmol) dissolved in THF was added dropwise. The reaction mixture was stirred at room temperature overnight. A saturated solution of K_2_CO_3_ was added, and the mixture was stirred for 2 h. Finally, the reaction mixture was filtered to obtain a white solid (6.86 g, 80%) corresponding to **G1** that was washed with water (3 × 20 mL). M.p: 238–240 °C. ^1^H-NMR (CDCl_3_, ppm) δ: 8.18 (s, 2H, NH), 8.03–8.01 (d, J = 7.4 Hz, 4H, o-Ph), 7.69–7.63 (m, 6H, m,p-Ph). ^13^C-NMR (CDCl_3_, ppm) δ: 164.9, 132.2, 131.9, 128.8, 127.7. The MS calculated for (C_14_H_12_N_2_O_2_) M+ 240.09 was found to be 240.98.

3,4,5-trimethoxy-N’-(3,4,5-trimethoxybenzoyl)benzohydrazide (**G2**): In a two-necked flask, THF (20 mL), hydrazine (0.70 g, 21.74 mmol) and Et_3_N (20 mL) were added at 0 °C under an inert atmosphere. Then, acid chloride (10.0 g, 43.47 mmol) dissolved in THF was added dropwise. The reaction mixture was stirred at room temperature overnight. A saturated K_2_CO_3_ solution was added, and the mixture was stirred for 2 h. Finally, the reaction mixture was filtered to obtain a white solid (6.85 g, 75%) corresponding to **G2** that was washed with water (3 × 20 mL). M.p: 245–247 °C. ^1^H-NMR (CDCl_3_, ppm) δ: 8.16 (s, 2H, NH), 7.17 (s, 4H, o-Ph), 3.91 (s, 18H, -OCH_3_) ^13^C-NMR (CDCl_3_, ppm) δ: 164.8, 153.1, 142.7, 128.6, 126.6, 60.6, 56.4. The MS calculated for (C_20_H_24_N_2_O_8_) M+ 420.15 was found to be 420.65.

N’-benzoyl-N,N’-dihexylbenzohydrazide (**G3**): A mixture of **G1** (5 g, 20.82 mmol), potassium tert-butoxide (2.71 g, 24.15 mmol), and 1-bromohexane (3.61 g, 21.86 mmol) was dissolved in methanol (50 mL). The reaction was heated at reflux for 12 h. The solvent was then removed under reduced pressure, and the residue was dissolved in CHCl_3_ and washed with H_2_O and brine. The organic phase was dried over MgSO4, the solvent was removed, and column chromatography was carried out while employing hexane/AcOEt 9:1 as the eluant, thus obtaining compound **G3** as a pale yellow solid (6.21 g, 73%). M.p: 202–204°C. ^1^H-NMR (CDCl_3_, ppm) δ: 8-03-8.01 (d, J = 7.4 Hz,, 4H, o-Ph), 7.68–7.63 (m, 6H, m,p-Ph), 4.62 (t, J = 7.0 Hz, 4H, N-CH_2_), 1.56–1.52 (t, J = 7.0 Hz, 4H, -CH_2_), 1.31–1.26 (m, 12H, -CH_2_), 0.90 (t, J = 7.0 Hz, 6H, -CH_3_) ^13^C-NMR (CDCl_3_, ppm) δ: 171.5, 137.9, 132.1, 128.9, 127.5, 44.6, 28.7, 27.4, 27.2, 22.6, 14.6. The MS calculated for (C_26_H_36_N_2_O_2_) M+ 408.28 was found to be 408.05.

## Figures and Tables

**Table 1:** CGC values for derivative **G1**.

Solvent	CGC (%wt.)
Dichloromethane	0.6
Ethanol	1.5
1-Pentanol	0.8
1-Propanol	0.8
2-Butanol	1.3
Acetone	1.2
Acetonitrile	1.5
Water	1.0
Ethyl acetate	0.6

**Table 2:** T_sol_ for gels of **G1** at 2% wt. and 1% wt.

Solvent	T_sol_ (°C) (2% wt.)	T_sol_ (°C) (1% wt.)
Dichloromethane	66	70
Ethanol	82	---
1-Pentanol	110	78
1-Propanol	62	63
2-Butanol	105	---
Acetone	94	---
Acetonitrile	93	---
Water	121	72
Ethyl acetate	132	74

## Data Availability

Not applicable.

## Supplementary Materials

The following supporting information can be downloaded at: https://www.mdpi.com/article/10.3390/gels9010026/s1. Tables S1–S9: Gelation test at different concentrations of **G1**–**G3**; Figures S1–S8: Rheology experiments for **G1**; Tables S10–S17: Crystal data for **G2**. Reference [40] is cited in the supplementary materials
