# Supplementary material for: Highly Thermally Resistant Bisamide Gelators as Pharmaceutical Crystallization Media
Source: Gels. 2022 Dec 29;9(1):26. doi: 10.3390/gels9010026 (PMC9858072; doi:10.3390/gels9010026)
Supplement: Supplementary file 1 [file gels-09-00026-s001.zip › gels-2035838-supplementary.pdf]

Supporting Information

Highly Thermally Resistant Bisamide Gelators as Pharmaceutical Crystallization Media

Iván Torres-Moya [1,2,*], Abelardo Sánchez [2], Basanta Saikia [3,4], Dmitry S. Yufit [3], Pilar Prieto [2], J. R. Carrillo [2] and Jonathan W. Steed [3,*]

[1] Department of Organic Chemistry, Faculty of Chemistry, University of Murcia, 30100 Murcia, Spain
[2] Department of Organic Chemistry, Faculty of Chemical Sciences and Technologies, University of Castilla La Mancha-IRICA, 13071 Ciudad Real, Spain; abelardo.sanchez@uclm.es (A.S.); mariapilar.prieto@uclm.es (P.P.); joseramon.carrillo@uclm.es (J.R.C.)
[3] Department of Chemistry, Durham University, South Road, Durham DH1 3LE, UK; bsaikia1@gmail.com (B.S.); d.s.yufit@durham.ac.uk (D.S.Y.)
[4] Department of Chemical Sciences, Tezpur University, Napaam-784028, India
* Correspondence: ivan.torres@um.es (I.T.-M.); jon.steed@durham.ac.uk (J.W.S.)

1. Gelation test

(S= Soluble, I= Insoluble, P= Precipitate, PG= Partial Gel, G= Gel)

Table S1. Gelation test for **G1** at a concentration of 2% wt.

Solvent	30 min	4h	24h	48h	72h
Chloroform	S	S	S	S	S
Dichloromethane	PG	PG	PG	G	G
Diethyl ether	I	I	I	S	S
Diethylene glycol	S	S	C	C	C
DMSO	S	S	S	S	S
Ethanol	S	S	G	G	G
Hexane	S	P	P	P	P
THF	S	P	P	P	P
1,4-dioxane	I	I	I	I	I
1-butanol	S	S	S	S	S
1-pentanol	G	G	G	G	G
1-propanol	PG	PG	PG	G	G
2-butanol	G	G	G	G	G
2-propanol	S	S	S	P	P
Acetone	G	G	G	G	G
Acetonitrile	G	G	G	G	G
Benzyl alcohol	S	S	S	S	S
Methanol	S	S	S	S	S
Cyclopentanone	S	S	S	P	P
Cyclohexane	S	S	S	P	P
Water	S	S	G	G	G
Diisopropylether	I	I	I	I	I
DMF	S	S	S	S	S
1,4-butanediol	S	S	S	S	S
Ethyl acetate	G	G	G	G	G
1,2-dibromoethane	I	I	I	I	I
Toluene	S	S	S	S	S
Nitromethane	S	S	S	S	S
o-Xylene	I	I	I	I	I
1,2,4-trichlorobenzene	I	I	I	I	I

Table S2. Gelation test for **G1** at a concentration of 1% wt.

Solvent	30 min	4h	24h	48h	72h
Chloroform	S	S	S	S	S
Dichloromethane	G	G	G	G	G
Diethyl ether	I	I	S	S	S
Diethylene glycol	S	S	C	C	C
DMSO	S	S	S	S	S
Ethanol	S	S	S	S	S
Hexane	S	P	P	P	P
THF	S	P	P	P	P
1,4-dioxane	I	I	I	I	I
1-butanol	S	S	S	S	S
1-pentanol	S	S	G	G	G
1-propanol	S	G	G	G	G
2-butanol	S	S	S	S	S
2-propanol	S	S	S	P	P
Acetone	S	S	S	S	S
Acetonitrile	S	S	S	S	S
Benzyl alcohol	S	S	S	S	S
Methanol	S	S	S	S	S
Cyclopentanone	S	S	S	P	P
Cyclohexane	S	S	S	P	P
Water	S	S	S	S	G
Diisopropylether	I	I	I	I	I
DMF	S	S	S	S	S
1,4-butanediol	S	S	S	S	S
Ethyl acetate	G	G	G	G	G
1,2-dibromoethane	I	I	I	I	I
Toluene	S	S	S	S	S
Nitromethane	S	S	S	S	S
o-Xylene	S	S	S	S	S
1,2,4-trichlorobenzene	S	S	S	S	S

Table S3. Gelation test for **G1** at a concentration of 0.5% wt.

Solvent	30 min	4h	24h	48h	72h
Chloroform	S	S	S	S	S
Dichloromethane	S	S	S	S	S
Diethyl ether	S	S	S	S	S
Diethylene glycol	S	S	C	C	C
DMSO	S	S	S	S	S
Ethanol	S	S	S	S	S
Hexane	S	P	P	P	P
THF	S	P	P	P	P
1,4-dioxane	I	I	I	I	I
1-butanol	S	S	S	S	S
1-pentanol	S	S	S	S	S
1-propanol	S	S	S	S	S
2-butanol	S	S	S	S	S
2-propanol	S	S	S	P	P
Acetone	S	S	S	S	S
Acetonitrile	S	S	S	S	S
Benzyl alcohol	S	S	S	S	S
Methanol	S	S	S	S	S
Cyclopentanone	S	S	S	P	P
Cyclohexane	S	S	S	P	P
Water	S	S	S	S	S
Diisopropylether	S	S	S	S	S
DMF	S	S	S	S	S
1,4-butanediol	S	S	S	S	S
Ethyl acetate	S	S	S	S	S
1,2-dibromoethane	S	S	S	S	S
Toluene	S	S	S	S	S
Nitromethane	S	S	S	S	S
o-Xylene	S	S	S	S	S
1,2,4-trichlorobenzene	S	S	S	S	S

Table S4. Gelation test for **G2** at a concentration of 2% wt.

Solvent	30 min	4h	24h	48h	72h
Chloroform	S	P	P	P	P
Dichloromethane	S	P	P	P	P
Diethyl ether	S	P	P	P	P
Diethylene glycol	S	S	S	S	S
DMSO	S	S	S	S	S
Ethanol	S	C	C	C	C
Hexane	S	P	P	P	P
THF	S	S	S	S	S
1,4-dioxane	S	P	P	P	P
1-butanol	S	P	P	P	P
1-pentanol	S	P	P	P	P
1-propanol	S	P	P	P	P
2-butanol	S	P	P	P	P
2-propanol	S	P	P	P	P
Acetone	S	S	S	S	S
Acetonitrile	S	P	P	P	P
Benzyl alcohol	S	P	P	P	P
Methanol	S	P	P	P	P
Cyclopentanone	S	P	P	P	P
Cyclohexane	S	P	P	P	P
Water	S	S	S	S	S
Diisopropylether	S	S	S	S	S
DMF	S	S	S	S	S
1,4-butanediol	S	S	S	S	S
Ethyl acetate	S	P	P	P	P
1,2-dibromoethane	S	S	S	S	S
Toluene	S	S	S	S	S
Nitromethane	S	P	P	P	P
o-Xylene	S	P	P	P	P
1,2,4-trichlorobenzene	S	P	P	P	P

Table S5. Gelation test for **G2** at a concentration of 1% wt.

Solvent	30 min	4h	24h	48h	72h
Chloroform	S	P	P	P	P
Dichloromethane	S	P	P	P	P
Diethyl ether	S	P	P	P	P
Diethylene glycol	S	S	S	S	S
DMSO	S	S	S	S	S
Ethanol	S	C	C	C	C
Hexane	S	P	P	P	P
THF	S	S	S	S	S
1,4-dioxane	S	P	P	P	P
1-butanol	S	P	P	P	P
1-pentanol	S	P	P	P	P
1-propanol	S	P	P	P	P
2-butanol	S	P	P	P	P
2-propanol	S	P	P	P	P
Acetone	S	S	S	S	S
Acetonitrile	S	P	P	P	P
Benzyl alcohol	S	P	P	P	P
Methanol	S	P	P	P	P
Cyclopentanone	S	P	P	P	P
Cyclohexane	S	P	P	P	P
Water	S	S	S	S	S
Diisopropylether	S	S	S	S	S
DMF	S	S	S	S	S
1,4-butanediol	S	S	S	S	S
Ethyl acetate	S	P	P	P	P
1,2-dibromoethane	S	S	S	S	S
Toluene	S	S	S	S	S
Nitromethane	S	P	P	P	P
o-Xylene	S	P	P	P	P
1,2,4-trichlorobenzene	S	P	P	P	P

Table S6. Gelation test for **G2** at a concentration of 0.5% wt.

Solvent	30 min	4h	24h	48h	72h
Chloroform	S	P	P	P	P
Dichloromethane	S	P	P	P	P
Diethyl ether	S	P	P	P	P
Diethylene glycol	S	S	S	S	S
DMSO	S	S	S	S	S
Ethanol	S	C	C	C	C
Hexane	S	P	P	P	P
THF	S	S	S	S	S
1,4-dioxane	S	P	P	P	P
1-butanol	S	P	P	P	P
1-pentanol	S	P	P	P	P
1-propanol	S	P	P	P	P
2-butanol	S	P	P	P	P
2-propanol	S	P	P	P	P
Acetone	S	S	S	S	S
Acetonitrile	S	P	P	P	P
Benzyl alcohol	S	P	P	P	P
Methanol	S	P	P	P	P
Cyclopentanone	S	P	P	P	P
Cyclohexane	S	P	P	P	P
Water	S	S	S	S	S
Diisopropylether	S	S	S	S	S
DMF	S	S	S	S	S
1,4-butanediol	S	S	S	S	S
Ethyl acetate	S	P	P	P	P
1,2-dibromoethane	S	S	S	S	S
Toluene	S	S	S	S	S
Nitromethane	S	P	P	P	P
o-Xylene	S	P	P	P	P
1,2,4-trichlorobenzene	S	P	P	P	P

Table S7. Gelation test for **G3** at a concentration of 2% wt.

Solvent	30 min	4h	24h	48h	72h
Chloroform	S	P	P	P	P
Dichloromethane	S	P	P	P	P
Diethyl ether	S	P	P	P	P
Diethylene glycol	S	S	S	S	S
DMSO	S	S	S	S	S
Ethanol	S	C	C	C	C
Hexane	S	P	P	P	P
THF	S	S	S	S	S
1,4-dioxane	S	P	P	P	P
1-butanol	S	P	P	P	P
1-pentanol	S	P	P	P	P
1-propanol	S	P	P	P	P
2-butanol	S	P	P	P	P
2-propanol	S	P	P	P	P
Acetone	S	S	S	S	S
Acetonitrile	S	P	P	P	P
Benzyl alcohol	S	P	P	P	P
Methanol	S	P	P	P	P
Cyclopentanone	S	P	P	P	P
Cyclohexane	S	P	P	P	P
Water	S	S	S	S	S
Diisopropylether	S	S	S	S	S
DMF	S	S	S	S	S
1,4-butanediol	S	S	S	S	S
Ethyl acetate	S	P	P	P	P
1,2-dibromoethane	S	S	S	S	S
Toluene	S	S	S	S	S
Nitromethane	S	P	P	P	P
o-Xylene	S	P	P	P	P
1,2,4-trichlorobenzene	S	P	P	P	P

Table S8. Gelation test for **G3** at a concentration of 1% wt.

Solvent	30 min	4h	24h	48h	72h
Chloroform	S	P	P	P	P
Dichloromethane	S	P	P	P	P
Diethyl ether	S	P	P	P	P
Diethylene glycol	S	S	S	S	S
DMSO	S	S	S	S	S
Ethanol	S	C	C	C	C
Hexane	S	P	P	P	P
THF	S	S	S	S	S
1,4-dioxane	S	P	P	P	P
1-butanol	S	P	P	P	P
1-pentanol	S	P	P	P	P
1-propanol	S	P	P	P	P
2-butanol	S	P	P	P	P
2-propanol	S	P	P	P	P
Acetone	S	S	S	S	S
Acetonitrile	S	P	P	P	P
Benzyl alcohol	S	P	P	P	P
Methanol	S	P	P	P	P
Cyclopentanone	S	P	P	P	P
Cyclohexane	S	P	P	P	P
Water	S	S	S	S	S
Diisopropylether	S	S	S	S	S
DMF	S	S	S	S	S
1,4-butanediol	S	S	S	S	S
Ethyl acetate	S	P	P	P	P
1,2-dibromoethane	S	S	S	S	S
Toluene	S	S	S	S	S
Nitromethane	S	P	P	P	P
o-Xylene	S	P	P	P	P
1,2,4-trichlorobenzene	S	P	P	P	P

Table S9. Gelation test for **G3** at a concentration of 0.5% wt.

Solvent	30 min	4h	24h	48h	72h
Chloroform	S	P	P	P	P
Dichloromethane	S	P	P	P	P
Diethyl ether	S	P	P	P	P
Diethylene glycol	S	S	S	S	S
DMSO	S	S	S	S	S
Ethanol	S	C	C	C	C
Hexane	S	P	P	P	P
THF	S	S	S	S	S
1,4-dioxane	S	P	P	P	P
1-butanol	S	P	P	P	P
1-pentanol	S	P	P	P	P
1-propanol	S	P	P	P	P
2-butanol	S	P	P	P	P
2-propanol	S	P	P	P	P
Acetone	S	S	S	S	S
Acetonitrile	S	P	P	P	P
Benzyl alcohol	S	P	P	P	P
Methanol	S	P	P	P	P
Cyclopentanone	S	P	P	P	P
Cyclohexane	S	P	P	P	P
Water	S	S	S	S	S
Diisopropylether	S	S	S	S	S
DMF	S	S	S	S	S
1,4-butanediol	S	S	S	S	S
Ethyl acetate	S	P	P	P	P
1,2-dibromoethane	S	S	S	S	S
Toluene	S	S	S	S	S
Nitromethane	S	P	P	P	P
o-Xylene	S	P	P	P	P
1,2,4-trichlorobenzene	S	P	P	P	P

2. Rheology experiments

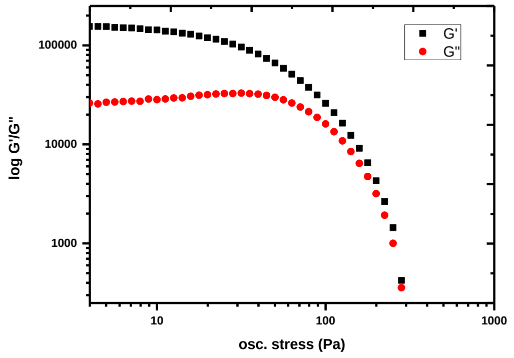

Figure S1. Stress sweep experiments at a concentration of 2% wt for **G1** in 2-butanol (s =316 Pa)

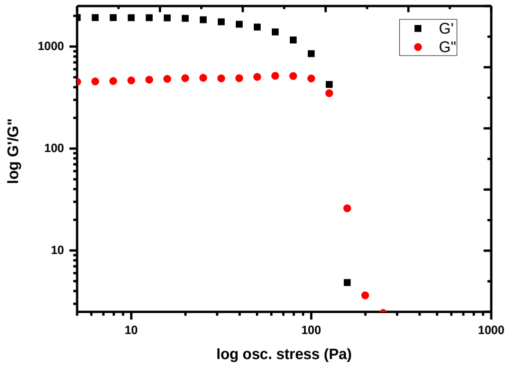

Figure S2. Stress sweep experiments at a concentration of 2% wt for **G1** in acetonitrile (s =133 Pa)

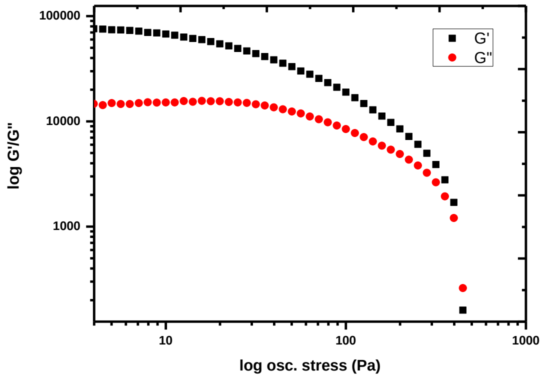

Figure S3. Stress sweep experiments at a concentration of 2% wt for **G1** in acetone (s=446 Pa)

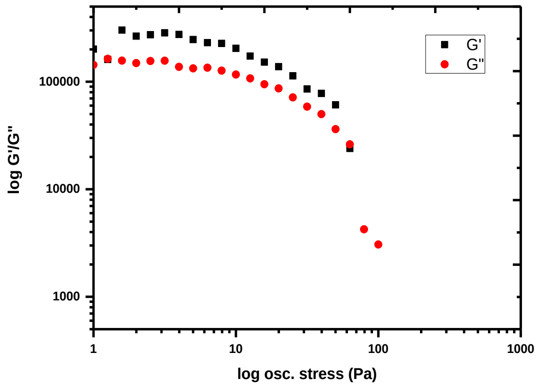

Figure S4. Stress sweep experiments at a concentration of 2% wt for **G1** in ethyl acetate (s =63 Pa)

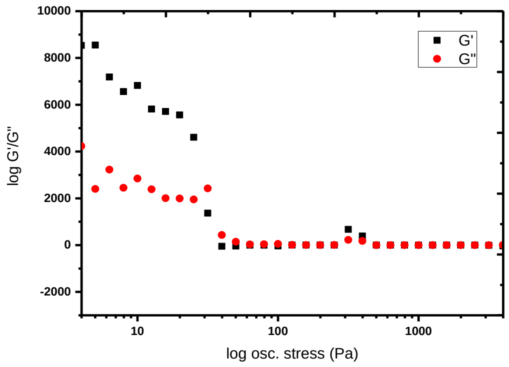

Figure S5. Stress sweep experiments at a concentration of 2% wt for **G1** in water (s =32Pa)

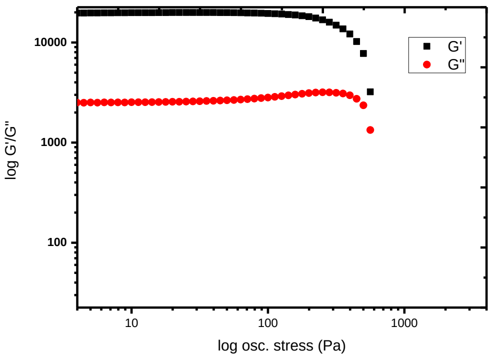

Figure S6. Stress sweep experiments at a concentration of 2% wt for **G1** in dichloromethane (s =631 Pa)

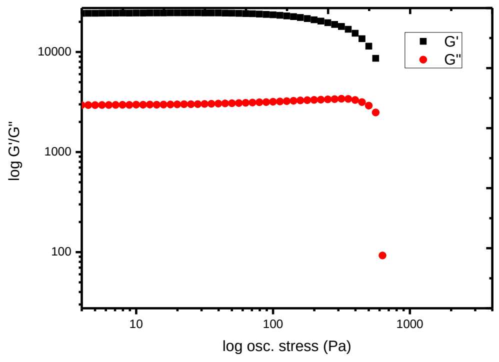

Figure S7. Stress sweep experiments at a concentration of 2% wt for **G1** in 1-propanol (s =631 Pa)

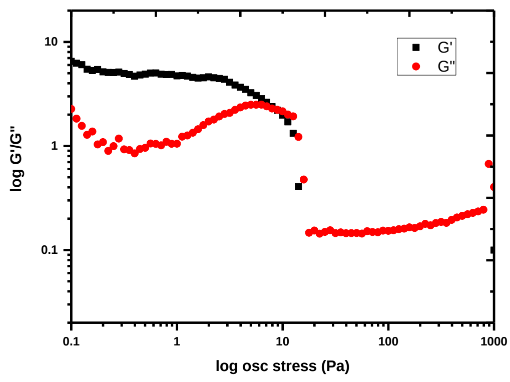

Figure S8. Stress sweep experiments at a concentration of 1% wt for **G1** in 1-pentanol (s =10 Pa)

3. Crystallization data of flufenamic acid

Crystals of flufenamic acid inside the gelator **G1**

a= 39.678 (9)

b= 5.066 (2)

g= 11.954 (3)

V= 2393 (2)

Orthorhombic P

Space group: $C_{2/c}$

Form III Polymorph [1]

References

[1] López-Mejías, V.; Kampf, J. W.; Matzger, A. J. *J. Am. Chem. Soc.* **2012**, 134, 24, 9872–9875.

4. **Crystal data of G2**

G2

**Table S10**. Crystal data and structure refinement for **G2**.	
Identification code	G2
Empirical formula	$C_{20}H_{24}N_2O_8$ x 2 H_2O
Formula weight	456.44
Temperature/K	120.0
Crystal system	monoclinic
Space group	$P2_1/c$
a/Å	15.8619(12)
b/Å	4.7691(4)
c/Å	13.7438(11)
α/°	90
β/°	96.490(3)
γ/°	90
Volume/Å^3	1033.01(14)
Z	2
ρ_{calc}g/cm^3	1.467
μ/mm^{-1}	0.118
F(000)	484.0
Crystal size/mm^3	0.22 × 0.15 × 0.02
Radiation	MoKα (λ = 0.71073)

2Θ range for data collection/°	5.17 to 57.976
Index ranges	-21 ≤ h ≤ 21, -6 ≤ k ≤ 6, -18 ≤ l ≤ 18
Reflections collected	15081
Independent reflections	2753 [R_{int} = 0.0650, R_{sigma} = 0.0582]
Data/restraints/parameters	2753/0/201
Goodness-of-fit on F^2	1.023
Final R indexes [I>=2σ (I)]	R_1 = 0.0461, wR_2 = 0.0927
Final R indexes [all data]	R_1 = 0.0847, wR_2 = 0.1043
Largest diff. peak/hole / e Å^{-3}	0.27/-0.32

Table S11. Fractional Atomic Coordinates (×10^4) and Equivalent Isotropic Displacement Parameters (Å^2×10^3) for **G2**. U_{eq} is defined as 1/3 of of the trace of the orthogonalised U_{IJ} tensor.

**Atom**	***x***	***y***	***z***	**U(eq)**
O1	9148.6(7)	3996(2)	4582.5(8)	16.6(3)
O2	7298.0(7)	-2084(2)	7512.3(8)	17.4(3)
O3	5993.8(6)	1187(2)	6804.8(8)	13.7(2)
O4	6040.2(6)	4145(2)	5171.4(8)	14.5(3)
N1	9620.4(8)	-152(3)	5208.4(10)	13.6(3)
C1	9025.1(9)	1852(3)	5053.9(11)	11.1(3)
C2	8225.2(9)	1425(3)	5499.0(11)	10.9(3)
C3	8169.3(9)	-373(3)	6286.1(11)	11.9(3)
C4	7415.9(9)	-504(3)	6713.0(11)	12.4(3)
C5	6717.2(9)	1114(3)	6341.1(11)	11.5(3)
C6	6770.5(9)	2795(3)	5520.8(11)	11.6(3)
C7	7530.1(9)	3019(3)	5111.1(11)	11.5(3)

C8	7974.3(10)	-3903(4)	7883.7(13)	17.9(4)
C9	5464.0(10)	-1258(4)	6638.1(13)	17.2(4)
C10	6064.0(11)	5844(4)	4316.3(12)	16.6(3)
O1W	10099.8(8)	4535(3)	3010.8(9)	18.8(3)

Table S12. Anisotropic Displacement Parameters (Å^2×10^3) for **G2**. The Anisotropic displacement factor exponent takes the form: $-2\pi^2[h^2a^{*2}U_{11}+2hka^*b^*U_{12}+\ldots]$.

Atom	U_{11}	U_{22}	U_{33}	U_{23}	U_{13}	U_{12}
O1	14.9(5)	14.4(6)	21.7(6)	5.6(5)	6.8(4)	1.1(5)
O2	16.8(6)	21.0(6)	15.5(6)	9.0(5)	6.8(4)	4.8(5)
O3	11.8(5)	14.3(5)	16.4(6)	-0.8(4)	8.0(4)	-1.3(4)
O4	10.7(5)	19.9(6)	13.3(6)	4.9(5)	2.9(4)	4.8(4)
N1	8.6(6)	12.8(7)	20.6(7)	3.4(6)	7.1(5)	0.2(5)
C1	11.9(7)	10.6(7)	10.6(7)	-1.2(6)	-0.3(6)	-0.4(6)
C2	11.1(7)	10.9(7)	11.2(7)	-2.9(6)	3.0(6)	-1.1(6)
C3	12.0(7)	11.9(7)	11.9(8)	0.3(6)	1.9(6)	1.8(6)
C4	15.5(7)	13.3(8)	8.9(7)	0.7(6)	3.4(6)	-0.6(6)
C5	11.3(7)	12.1(7)	11.6(7)	-2.6(6)	4.0(6)	-2.0(6)
C6	11.4(7)	11.1(7)	12.1(8)	-2.3(6)	1.0(6)	1.1(6)
C7	13.4(8)	10.7(7)	10.8(8)	-0.3(6)	3.0(6)	0.1(6)
C8	17.1(8)	19.8(9)	17.1(9)	7.6(8)	2.7(7)	2.6(7)
C9	15.8(8)	14.7(8)	22.4(9)	-0.9(7)	7.4(7)	-3.2(7)
C10	14.5(8)	19.8(9)	15.5(8)	6.5(7)	1.7(6)	3.0(7)
O1W	22.2(6)	17.2(6)	17.9(6)	2.8(5)	7.1(5)	4.3(5)

**Table S13**. Bond Lengths for **G2**.						
**Atom**	**Atom**	**Length/Å**		**Atom**	**Atom**	**Length/Å**
O1	C1	1.2379(18)		N1	C1	1.3435(19)
O2	C4	1.3623(18)		C1	C2	1.483(2)
O2	C8	1.4285(19)		C2	C3	1.391(2)
O3	C5	1.3747(17)		C2	C7	1.395(2)
O3	C9	1.4404(19)		C3	C4	1.391(2)
O4	C6	1.3643(17)		C4	C5	1.399(2)
O4	C10	1.4315(19)		C5	C6	1.394(2)
N1	N1[1]	1.398(2)		C6	C7	1.390(2)

[1]2-X,-Y,1-Z

**Table S14**. Bond Angles for **G2**.								
**Atom**	**Atom**	**Atom**	**Angle/˚**		**Atom**	**Atom**	**Atom**	**Angle/˚**
C4	O2	C8	117.27(12)		O2	C4	C3	124.59(14)
C5	O3	C9	114.10(12)		O2	C4	C5	115.12(13)
C6	O4	C10	117.12(11)		C3	C4	C5	120.27(14)
C1	N1	N1[1]	118.93(17)		O3	C5	C4	120.86(13)
O1	C1	N1	121.36(13)		O3	C5	C6	119.38(13)
O1	C1	C2	121.50(13)		C6	C5	C4	119.67(13)
N1	C1	C2	117.12(13)		O4	C6	C5	115.43(12)
C3	C2	C1	122.55(13)		O4	C6	C7	124.16(14)
C3	C2	C7	121.07(13)		C7	C6	C5	120.41(14)
C7	C2	C1	116.34(14)		C6	C7	C2	119.16(14)
C4	C3	C2	119.28(14)					

[1]2-X,-Y,1-Z

**Table S15**. Hydrogen Bonds for **G2**.						
**D**	**H**	**A**	**d(D-H)/Å**	**d(H-A)/Å**	**d(D-A)/Å**	**D-H-A/°**
N1	H1	O1[1]	0.88(2)	2.23(2)	2.9905(18)	144.6(19)
O1W	H1WA	O1W[2]	0.86(2)	1.91(2)	2.7672(13)	176(2)
O1W	H1WB	O1	0.88(3)	1.92(3)	2.7830(17)	167(2)

[1]+X,-1+Y,+Z; [2]2-X,1/2+Y,1/2-Z

**Table S16**. Selected Torsion Angles for **G2**.										
**A**	**B**	**C**	**D**	**Angle/°**		**A**	**B**	**C**	**D**	**Angle/°**
N1[1]	N1	C1	O1	-2.7(3)		C5	C4	O2	C8	176.27(14)
C2	C1	N1	N1[1]	179.23(16)		C5	C6	O4	C10	-178.65(14)
C3	C2	C1	O1	-157.26(14)		C6	C5	O3	C9	106.39(16)
C3	C2	C1	N1	20.9(2)		C7	C2	C1	O1	20.2(2)
C3	C4	O2	C8	-5.5(2)		C7	C2	C1	N1	-161.67(14)
C4	C5	O3	C9	-77.18(18)		C7	C6	O4	C10	1.4(2)

[1]2-X,-Y,1-Z

**Table S17**. Hydrogen Atom Coordinates (Å×10^4) and Isotropic Displacement Parameters (Å^2×10^3) for **G2**.				
**Atom**	***x***	***y***	***z***	**U(eq)**
H1	9476(14)	-1900(50)	5307(16)	44(6)
H3	8637(11)	-1440(40)	6535(12)	15(4)
H7	7590(10)	4230(40)	4587(13)	15(4)
H8A	8161(10)	-5090(40)	7383(13)	16(4)
H8B	7731(11)	-5000(40)	8381(14)	26(5)
H8C	8456(12)	-2830(40)	8175(14)	26(5)
H9A	4988(11)	-920(40)	7054(13)	21(5)

H9B	5254(11)	-1340(40)	5927(15)	28(5)
H9C	5787(11)	-2890(40)	6809(12)	15(4)
H10A	6475(10)	7380(40)	4455(12)	14(4)
H10B	6264(11)	4740(40)	3762(14)	21(5)
H10C	5483(11)	6530(40)	4146(12)	16(4)
H1WA	10035(13)	6130(50)	2717(16)	40(7)
H1WB	9741(15)	4510(50)	3454(19)	53(7)
